# Severe pneumonia with empyema due to multiple anaerobic infections: case report and literature review

**DOI:** 10.3389/fmed.2024.1435823

**Published:** 2024-08-14

**Authors:** Fangyu Yu, Xiaojing Zhang, Yujiao Li, Wei Gai, Yafeng Zheng, Xudong Cai

**Affiliations:** ^1^Department of ICU, Ningbo Municipal Hospital of Traditional Chinese Medicine (TCM), Affiliated Hospital of Zhejiang Chinese Medical University, Ningbo, China; ^2^WillingMed Technology (Beijing) Co., Ltd, Beijing, China; ^3^Department of Nephrology, Ningbo Municipal Hospital of Traditional Chinese Medicine (TCM), Affiliated Hospital of Zhejiang Chinese Medical University, Ningbo, China

**Keywords:** severe pneumonia, empyema, anaerobic, mixed infection, metagenomic next generation sequencing

## Abstract

**Background:**

Cases of severe pneumonia complicated by empyema due to normal anaerobic flora from the oral cavity are infrequent. Diagnosing anaerobic infections through conventional microbiological test (CMT) is often challenging.

**Case presentation:**

This study describes the case of a 67-year-old man, bedridden long-term, who developed severe pneumonia with empyema caused by multiple anaerobic bacterial infections. The patient was hospitalized with a 5-day history of cough, sputum and fever, accompanied by a 2-day history of dyspnea. Despite CMT, the specific etiology remained elusive. However, metagenomic next-generation sequencing (mNGS) identified various anaerobic bacteria in bronchoalveolar lavage fluid (BALF), blood and pleural effusion. The patient was diagnosed with a polymicrobial infection involving multiple anaerobic bacteria. Following treatment with metronidazole and moxifloxacin, the patient’s pulmonary symptoms improved.

**Conclusion:**

mNGS serves as a valuable adjunctive tool for diagnosting and managing patients whose etiology remains unidentified following CMT.

## 1 Introduction

Empyema refers to the accumulation of purulent exudate in the pleural cavity due to invasion by pathogenic bacteria, a common complication of pneumonia. In recent years, there has been a global rise in the incidence of pneumonia complicated by empyema. Common causative agents include aerobic bacteria such as *Streptococcus pneumoniae*, *Staphylococcus aureus*, and *Klebsiella pneumoniae*, as well as anaerobic bacteria like *Bacteroides* and *Peptostreptococcus* (1, 2). Conversely, normal oral flora rarely lead to pneumonia or empyema and are challenging to identify through conventional microbiological test (CMT). However, isolated cases of severe pneumonia with empyema caused by single or dual oral anaerobic bacteria have been increasing reported (3–5), while infections involving multiple anaerobic species remain uncommon (6). Here, we report a case of pneumonia complicated by empyema in a patient admitted with a 5-day history of cough, sputum production, and fever, alongside 2 days of dyspnea. Metagenomic next generation sequencing (mNGS) confirmed a complex oral anaerobic infection as the etiology. This case underscores the critical role of advanced detection techniques in clinical practice.

## 2 Case presentation

A 67-year-old male patient was admitted to the hospital on February 26, 2024, presenting with a 5 day history of cough, sputum production, and fever, along with 2 days of dyspnea. Since Feb-22, the patient had developed an unprovoked cough, sputum and fever peaking at 38°C, without other notable symptoms. Administration of antipyretic drugs initially reduced the fever, which subsequently fluctuated between 37.3 and 37.5°C. By Feb-24, the patient began experiencing chest tightness and exacerbated dyspnea, especially at rest. These symptoms worsened by Feb-26, necessitating hospitalization at a local facility, Followed by transfer to our emergency department due to the severity of his condition. On admission, the patient presented with apathy, decreased peripheral blood oxygen level, and required emergency tracheal intubation before transfer to the intensive care unit (ICU). In 2005, the patient suffered a brainstem hemorrhage from a traffic accident, resulting in left limb hemiplegia and 2 years of bed rest. He had been in long-term nursing home care before hospitalization, suggesting nursing-home acquired pneumonia (NHAP). However, no definitive etiological examination results were provided by family members, nor was there information on a history of recurrent aspiration.

**FIGURE 1:**
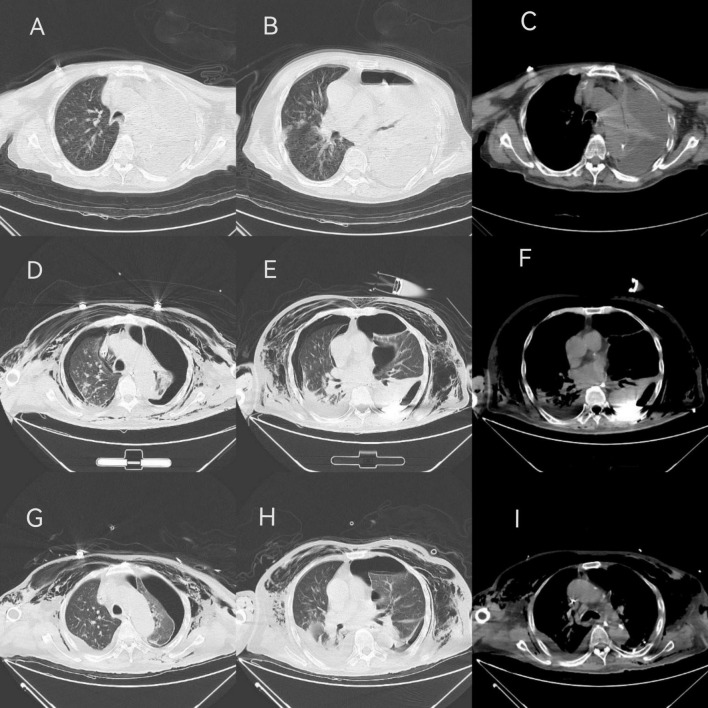
CT findings in different periods. **(A–C)** On admiration, the patient showed left pleural effusion, air accumulation, left lung atelectasis, local compactness, right lung infection, a small amount of pleural effusion on the right. **(D–F)** On Mar-01, the CT scan indicated bilateral liquid pneumothorax, local fluid accumulation on the left side showed a high-density shadow, two lungs atelectasis, local consolidation, two lungs infection, left lung consolidation and atelectasis improved. **(G–I)** On Mar-05, bilateral liquid pneumothorax still can be found, but left attasis slightly improved.

Upon admission to our hospital, the patient exhibited a temperature of 37.7°C, heart rate of 111 times/min, respiratory rate was 22 breaths/min, blood pressure was 91/61 mmHg, and oxygen saturation (SpO2%) of 95%. The patient was in a shallow coma, intubated and mechanically ventilated. Physical examination revealed moist rales in the right lung, diminished breath sounds in the left lung, normal heart rhythm, distended abdomen, soft without edema in the limbs, cool, moist skin on the left lower limb, intact left dorsal foot artery, and negative bilateral Babinski’s sign. Laboratory findings included elevated white blood cell (WBC, 18.2 × 10^9^/L), neutrophil percentage (N%, 94.6%), C-reactive protein (CRP, 293.9 mg/L), procalcitonin (PCT, 28.42 ng/mL), and creatinine (155 μmol/L), with significantly decreased albumin (21.1 g/L). There were no evidence of HIV or other immunosuppressive conditions. Abdominal ultrasound revealed dense liver echogenicity, gallbladder polyp, right kidney cyst, and multiple stones. Chest CT showed left pleural effusion, pneumothorax, left lung atelectasis, focal consolidation, and infection in the right lung with a small right-sided pleural effusion (Figures 1A–C). The initial diagnosis was severe pneumonia with empyema, with a SOFA score of 17. Detailed SOFA scores throughout the clinical course were listed in Supplementary Table 1. Upon ICU admission, a closed drainage puncture of the left chest was performed for purulent drainage. Empirically, meropenem (1.0 g, q8h+) and tigecycline (50 mg, q12h, ivgtt) was given for anti-infection treatment, as well as methylprednisolone (40 mg, q12h) for intensive anti-inflammatory therapy and stress relief.

Further etiological tests included antibody and nucleic acid testing for COVID-19, respiratory virus antibody panels (respiratory syncytial virus, adenovirus, parainfluenza virus), influenza A + B virus antigen, routine peripheral venous blood culture, midstream urine culture, thoracic drainage fluid culture, (1, 3)-β-D-glucan (G) and galactomannan (GM) test, all yielding negative results. Sputum and BALF cultures identified *Candida albicans*, and methicillin-susceptible *Staphylococcus aureus*, which did not fully correspond with the clinical symptoms.

**FIGURE 2:**
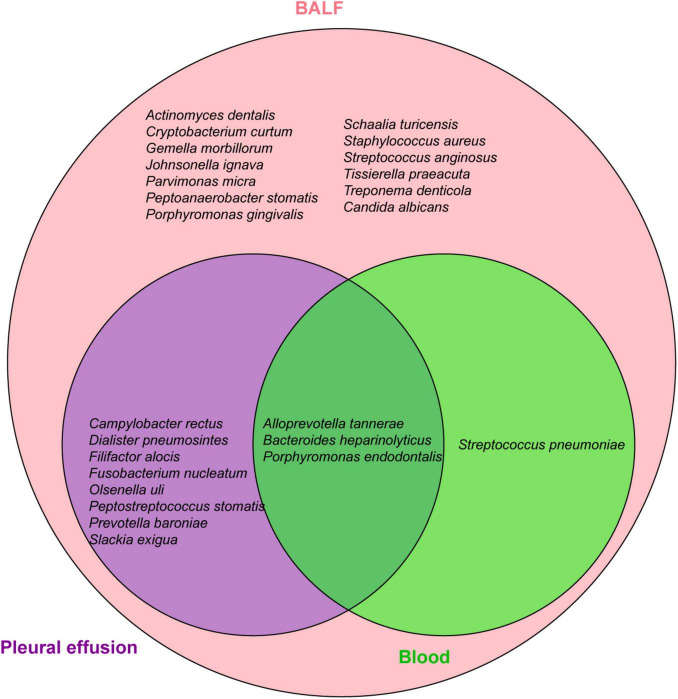
Distribution of pathogens detected by mNGS in BALF, blood and pleural effusion.

To identify the etiology, mNGS of blood, pleural effusion and bronchoalveolar lavage fluid (BALF) were performed on Feb-28 following family consent. The mNGS procedure followed previous report (7, 8). Both DNA and RNA were extracted from the samples, and libraries were prepared for sequencing using a 50 bp single-end sequencing kit on the MGISEQ-200 platform (MGI Technology). Raw FASTQ-format data underwent Fastq for quality control and evaluation. High-quality sequencing reads were aligned against the human reference genome GRCh37 (hg19) using Bowtie2 v2.4.3 to remove human host sequences. The remaining sequences were then compared against the NCBI GenBank database using Kraken2 v2.1.0 to annotate pathogen genomes and identify pathogens present in the samples. Pathogens were identified based on the specific reads per ten million (RPTM value). For virus detection, an RPTM value ≥ 3 was used as the threshold, while bacteria and fungi required an RPTM ≥ 8 for positive identification. Anaerobic bacteria, primarily oral colonizers, including *Parvimomas micra*, *Peptostreptococcus stomatis*, *Olsenella uli*, and *Slackia exigua*, were identified on Mar-01 (Figure 2). Relative pathogen abundance is detailed in Supplementary Table 2. Detection of the same pathogen in multiple sites indicates potential disseminated infection, and combined with the highest abundance of the anaerobic bacteria in BALF, pleural fluid, and blood, an anaerobic infection is strongly suspected in this patient. Thus, the antibiotic regimen was adjusted to metronidazole (500 mg, q6h+) and moxifloxacin (0.4 g, qd). Subsequent CT scans demonstrated bilateral pleural effusions, denser left sided effusion, atelectasis in both lungs, focal consolidation, and improved left lung consolidation and atelectasis compared to the previous one (Figures 1D–F).

**FIGURE 3:**
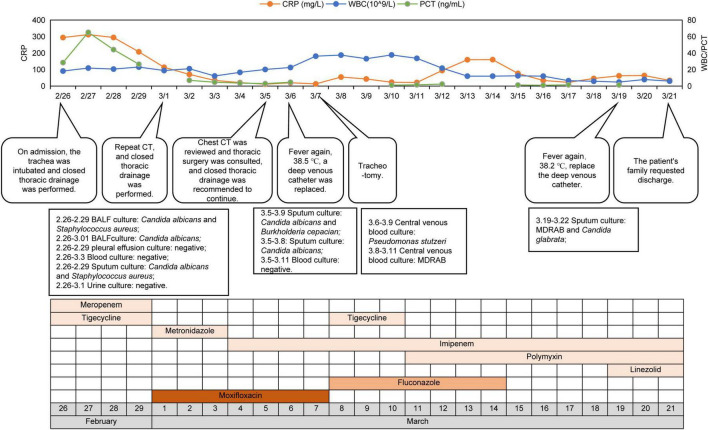
The clinical course of the patient. CRP, C-reactive protein; WBC, white blood cell; MDRAB, multidrug-resistant *Acinetobacter baumannii*.

Following antibiotic adjustment, WBC, CRP, and PCT levels declined significantly, accompanied by gradual reduction in vasopressor requirements, indicating clinical improvement. On Mar-04, following infectious disease expert consultation, meropenem was substituted with imipenem for anaerobic bacteria. By Mar-05, drainage from the left lung was satisfactory, with improved atelectasis compared to previous scans (Figures 1G–I). However, subsequent fever fluctuations and increased WBC and CRP levels prompted further adjustments to the antibiotic regimen: imipenem (1 g, q8h), metronidazole (500 mg, q6h), and subsequently polymyxin B (750000 U, q12h) and aerosolized colistin (250000 U, q12h), due to suspected bloodstream infection. Despite ongoing treatment, family decision led to discharge on Mar-22 with tracheotomy and ventilator support. The patient’s treatment course is detailed in Figure 3.

## 3 Discussion

The majority of complex empyema cases arise subsequent to pneumonia. During pathogenesis, pathogens must breach pulmonary defense mechanisms, spread via the respiratory tract and alveoli, and ultimately reach the pleura. The high oxygen tension in the respiratory tract effectively inhibits anaerobic bacterial growth, through it does not entirely prevent it; infection typically occurs through accidental inhalation of oral or gastric contents (9). At present, empyema caused by oral or gastrointestinal anaerobe is common, but cases involving rare or multiple anaerobic infections, or severe infections, are infrequent. Etiological detection and culture of rare anaerobic bacteria pose challenges, often necessitating mNGS or 16s rRNA analysis.

Out study presents a case of severe pneumonia with empyema caused by various oral anaerobes identified by mNGS in a long-term bedridden patient. A PubMed search using keywords “Pleural Empyema” and “anaerobic” yielded clinical case reports of pneumonia and anaerobic thoracic infections (Supplementary Table 3). Most cases involved male smokers, some with documented oral infections, and a few with anatomical anomalies such as pleural fistula, none had significant immunosuppression. Most patients presented solely with empyema, rarely concurrent with pneumothorax. Treatment primarily involved closed chest drainage of pus, with some undergoing thoracoscopic surgery or pleural dissection; mechanical ventilation was seldom required, and prognosis was generally favorable.

The patient in this study had no history of smoking or alcohol abuse, no did he have clear oral infection history or related anatomical abnormalities. The patient suffered from extensive and severe lung infection, predominantly affecting the entire left lung. This led to the accumulation of a significant amount of pleural pus, resulting in left lung atelectasis and compression of the right lung, necessitating endotracheal tube, mechanical ventilation and vasoactive agents to maintain vital signs. The patient also displayed signs of systemic infection, prompting the use of vasoactive medications and endotracheal intubation for ventilator support. Throughout the disease course, the patient developed severe pneumothorax and subcutaneous emphysema. In addition to drainage of pleural fluid and two closed chest drains to relieve pneumothorax symptoms, the patient lung re-expansion was suboptimal, and recurrent fever persisted. The possibility of bloodstream infections was considered, and surgical interventions were deliberated but ultimately withheld due to the patient’s poor overall condition and the family’s wishes. The prognosis was grim, and efforts were made to minimize sedation and analgesia to facilitate awakening before discharge. However, the patient remained unconsciousness and unable to breathe spontaneously off the ventilator.

Anaerobic bacterial infections mainly involve single or dual types of oral or intestinal anaerobes, including *Parvimonas micra*, *Fusobacterium*, *Staphylococcus*, *Actinomyces*, and *Peptostreptococcus* (Supplementary Table 3). The anaerobic profile in our patient were complex; mNGS identified distinct bacteria in blood, BALF, and pleural effusion, predominantly oral anaerobes and opportunistic pathogens. Blood reads were minimal, whereas BALF and pleural effusion were dominated by *Peptostreptococcus stomatis*, *Parvimonas micra*, *Olsenella uli*, particularly *Slackia exigua* with more than 10,000 reads, and *P. stomatis* with over 50,000 reads in the pleural effusion (Figure 2). Apart from *P. micra*, implicated in prior anaerobic empyema cases (10, 11), reports on *P. stomatis*, *O. uli*, and *S. exigua* are scarce. *P. micra*, a component of the normal flora in the oral cavity, and the gastrointestinal tract mucous, skin, and female reproductive tract (3), rarely causes pneumonia or empyema (12), but can lead to bacteremia (13). *P. stomatis*, widely distributed in the human oral cavity, upper respiratory tract, gastrointestinal tract, female genitourinary system, and skin, is part of the normal flora. No reports of *P. stomatis*-associated empyema have been reported, although other *Peptostreptococcus* species have caused empyema with atypical infection symptoms and possible chest pain (14–16). *O. uli* is a common member of the microbiota associated with primary endodontic infections and can also be isolated from patients with local oral or gastrointestinal infections (17–19), but cases of lung infections are rare. Yan et al. reported the first case of *O. uli* lung infection (4). The patient had a 30-year history of smoking but had quit 10 years prior. The patient developed a cough with bloody sputum without an obvious cause. CT findings indicated bilateral pneumonia, pyogenic necrosis of the right lower lobe, empyema of the right thoracic capsule, and bilateral emphysema. Treatment with ornidazole combined with ceftazidime resulted in significant improvement in lung lesions and pleural effusion. *S. exigua* is an obligate anaerobic bacterium associated with dental infections, but rarely causes extraneous infections. However, cases of pulmonary and bloodstream infections have been reported in recent years. Roingeard et al. performed non-invasive ventilation tracheotomy on a 29-year-old man with a history of severe neurological deficit due to Ravine syndrome, who was hospitalized with fever (20). The patient was found to have a large abscess in his left lung, and a localized effusion in the left pectoral muscle. Pulmonary abscess culture confirmed the presence of *S. exigua*, which responded to treatment with amoxicillin/clavulanic acid. Three cases of *S. exigua* bacteremia were reported, including one community-acquired bacteremia associated with pleural empyema and a postoperative intraperitoneal abscess in a 73-year-old man with primary intestinal diffuse large B-cell lymphoma (Supplementary Table 3), and one case associated with pyometra (21). These studies indicate that anaerobic bacteria can not only cause lung infections but also lead to bloodstream infections in patients with disease progression.

In this case, the patient had obvious pulmonary symptoms initially. Following the diagnosis of anaerobic bacterial infection, targeted medication led to improvement in the pulmonary symptoms. However, the patient’s condition subsequently deteriorated, and multidrug-resistant *Acinetobacter baumannii* (MDRAB) was detected in the blood, raising suspicion of bloodstream infection. The discontinuation of mNGS testing prevented accurate determination of whether anaerobic bloodstream infection was complicating the patient’s condition. This case underscores the importance of preventing anaerobic bacteria from spreading beyond the pulmonary infection site when it becomes severe.

In our current clinical practice, microbial diagnosis still heavily relies on conventional bacterial culture (22). However, bacteriological study of lung abscesses encounters significant challenges in sampling and culturing, particularly in avoiding oral contamination and exposure to oxygen. Moreover, bacterial growth conditions *in vivo* differ markedly from those in laboratory media, make it difficult to replicate the *in vivo* environment accurately. These factors, coupled with pathogen complexity and empirical antibiotics use, result in a lower sensitivity of conventional culture (23, 24). Consequently, the proportion of culture-negative samples exceeds that in actual situations. Additionally, 16S rRNA sequencing and Matrix-Assisted Laser Desorption Ionization Time of Flight Mass Spectrometry (MALDI-TOF MS) were also important tools for pathogen identification. While 16S rRNA sequencing rapidly and accurately identifies microorganisms at the genus level, its ability to differentiate between species or subspecies is limited (25). MALDI-TOF MS, a spectroscopic method reliant on a comprehensive database, offers rapid (less than 1 h) identification and high discriminatory power, particularly valuable for characterizing rare bacteria traditionally challenging to identify using routine methods (26). However, the database limitations of MALDI-TOF MS in anaerobic organism identification significantly impact MS sequencing data analysis, particularly in discerning mixed strains. Additionally, MALDI-TOF MS identification relies on bacteria culture, which as mentioned earlier, exhibits lower sensitivity. In our case, routine peripheral blood and pleural effusion culture yielded negative results, while *Candida albicans* and *Staphylococcus aureus* detected by sputum and BALF culture failed to explain the patient’s severe empyema. Despite obtaining cultures prior to antibiotic administration, the results were unsatisfactory. In such scenarios, mNGS, free from culture dependence and capable of detecting all nucleic acids without bias, appears optimal for identifying etiology (27). Although mNGS has limitations, such as generating a large number of sequences matching multiple microorganisms, resulting in false negatives and positives, and its high cost (28), its use remain crucial for etiological diagnosis in cases of culture-negative specimens post–antibiotic treatment, suspected uncommon pathogens infections.

In conclusion, pathogen detection in pneumonia and empyema patients infected by anaerobic bacteria remains challenging, even with pre-antibiotic culture samples. When suspicion of rare pathogen infection arises or when clinical etiological test results are negative or incongruent with symptoms, mNGS proves invaluable for timely and accurate pathogen identification, enabling targeted antimicrobial treatment in clinical practice.

## Data availability statement

The raw data supporting the conclusions of this article will be made available by the authors, without undue reservation.

## Ethics statement

The studies involving humans were approved by the Ethics committee of Ningbo Municipal Hospital of T.C.M. The studies were conducted in accordance with the local legislation and institutional requirements. The participants provided their written informed consent to participate in this study. Written informed consent was obtained from the individual(s) for the publication of any potentially identifiable images or data included in this article.

## Author contributions

FY: Conceptualization, Data curation, Formal analysis, Investigation, Visualization, Writing – original draft, Writing – review and editing. XZ: Formal analysis, Visualization, Writing – original draft, Writing – review and editing. YL: Writing – original draft, Writing – review and editing. WG: Writing – review and editing. YZ: Writing – review and editing. XC: Project administration, Visualization, Writing – review and editing.

## References

[1] Addala D Bedawi E Rahman N. Parapneumonic effusion and empyema. *Clin Chest Med.* (2021) 42:637–47. 10.1016/j.ccm.2021.08.001 34774171

[2] Kuryłek A Stasiak M Kern-Zdanowicz I. Virulence factors of *Streptococcus anginosus* - a molecular perspective. *Front Microbiol.* (2022) 13:1025136. 10.3389/fmicb.2022.1025136 36386673 PMC9643698

[3] Yu Q Sun L Xu Z Fan L Du Y. Severe pneumonia caused by *Parvimonas micra*: A case report. *BMC Infect Dis.* (2021) 21:364. 10.1186/s12879-021-06058-y 33865326 PMC8052845

[4] Yan Y Li H Li S Liu S Jia N Liu Y Olsenella uli-induced pneumonia: A case report. *Ann Clin Microbiol Antimicrob.* (2022) 21:9. 10.1186/s12941-022-00499-2 35232448 PMC8889775

[5] Duan Y Feng W Shen Y Li Y Li N Chen X Severe pneumonia with empyema caused by *Parvimonas micra* and *Streptococcus constellatus* co-infection: A case report. *J Int Med Res.* (2023) 51:3000605231210657. 10.1177/03000605231210657 37994021 PMC10666820

[6] Duport P Miltgen G Kebbabi C Belmonte O Coolen-Allou N Allyn J First case of pleural empyema and pulmonary abscess caused by *Eggerthia catenaformis*. *Anaerobe.* (2018) 50:9–11. 10.1016/j.anaerobe.2018.01.006 29371096

[7] Chen H Zheng Y Zhang X Liu S Yin Y Guo Y Clinical Evaluation of cell-free and cellular metagenomic next-generation sequencing of infected body fluids. *J Adv Res.* (2023) 55:119–29. 10.1016/j.jare.2023.02.018 36889461 PMC10770109

[8] Wu C Yu X Gai W Liu Y Qi Y Zheng Y Diagnostic value of plasma and blood cells metagenomic next-generation sequencing in patients with sepsis. *Biochem Biophys Res Commun.* (2023) 683:149079. 10.1016/j.bbrc.2023.10.011 37871447

[9] Allewelt M. Aspiration pneumonia and primary lung abscess: Diagnosis and therapy of an aerobic or an anaerobic infection? *Expert Rev Respir Med.* (2007) 1:111–9. 10.1586/17476348.1.1.111 20477271

[10] Gumbs S Kwentoh I Atiku E Gikunda W Safavi A. *Parvimonas micra*: A rare cause of pleural empyema with Covid-19 Co-infection. *Cureus.* (2024) 16:e51998. 10.7759/cureus.51998 38205082 PMC10777265

[11] Vilcarromero S Small M Lizarzaburu A Rivadeneyra-Rodriguez A. Pleural empyema by *Parvimonas micra* in an immunocompetent patient: A case report. *Rev Peru Med Exp Salud Publica.* (2023) 40:99–104. 10.17843/rpmesp.2023.401.11956 37377244 PMC10953646

[12] Shimizu K Horinishi Y Sano C Ohta R. Infection route of *Parvimonas micra*: A case report and systematic review. *Healthcare (Basel).* (2022) 10:1727. 10.3390/healthcare10091727 36141340 PMC9498800

[13] Yamada K Taniguchi J Kubota N Kawai T Idemitsu R Inoshima N Empyema and bacteremia caused by *Parvimonas micra*: A case report. *Respir Med Case Rep.* (2023) 45:101892. 10.1016/j.rmcr.2023.101892 37577121 PMC10413192

[14] Gülmez D Alp S Topeli Iskit A Akova M Hasçelik G. [Pneumonia caused by *Fusobacterium necrophorum*: Is lemierre syndrome still current?]. *Mikrobiyol Bul.* (2011) 45:729–34. 22090304

[15] Kikuchi N Nomura A Endo T Sekizawa K. Anaerobic bacterial empyema accompanying intrathoracic gas formation in anorexia nervosa. *Int J Eat Disord.* (2006) 39:621–3. 10.1002/eat.20275 16752426

[16] Martinez K Mangat G Sherwani N Glover D Silver M. Veillonella intrapulmonary abscess with empyema. *Cureus.* (2023) 15:e45210. 10.7759/cureus.45210 37842426 PMC10576213

[17] Dewhirst F Paster B Tzellas N Coleman B Downes J Spratt D Characterization of novel human oral isolates and cloned 16s RDNA sequences that fall in the family coriobacteriaceae: Description of *Olsenella* Gen. Nov., Reclassification of *Lactobacillus uli* as *Olsenella uli* Comb. Nov. and description of *Olsenella profusa* Sp. Nov. *Int J Syst Evol Microbiol.* (2001) 51:1797–804. 10.1099/00207713-51-5-1797 11594611

[18] Lau S Woo P Fung A Chan K Woo G Yuen K. Anaerobic, non-sporulating, gram-positive bacilli bacteraemia characterized by 16s RRNA gene sequencing. *J Med Microbiol.* (2004) 53:1247–53. 10.1099/jmm.0.45803-0 15585505

[19] Bahrani-Mougeot F Paster B Coleman S Ashar J Barbuto S Lockhart P. Diverse and novel oral bacterial species in blood following dental procedures. *J Clin Microbiol.* (2008) 46:2129–32. 10.1128/jcm.02004-07 18434561 PMC2446827

[20] Roingeard C Jaubert J Guilleminault L. A large and unusual lung abscess with positive culture to *Slackia exigua*. *Int J Infect Dis.* (2015) 40:37–8. 10.1016/j.ijid.2015.09.015 26432408

[21] Lim K Son J Moon SY. A case of *Slackia exigua* bacteremia associated with pyometra in a patient with poor dentition. *Anaerobe.* (2022) 73:102477. 10.1016/j.anaerobe.2021.102477 34780915

[22] Lasken R McLean J. Recent advances in genomic DNA sequencing of microbial species from single cells. *Nat Rev Genet.* (2014) 15:577–84. 10.1038/nrg3785 25091868 PMC4454502

[23] Saglani S Harris K Wallis C Hartley J. Empyema: The use of broad range 16s RDNA PCR for pathogen detection. *Arch Dis Child.* (2005) 90:70–3. 10.1136/adc.2003.042176 15613518 PMC1720100

[24] Le Monnier A Carbonnelle E Zahar J Le Bourgeois M Abachin E Quesne G Microbiological diagnosis of empyema in children: Comparative evaluations by culture, polymerase chain reaction, and pneumococcal antigen detection in pleural fluids. *Clin Infect Dis.* (2006) 42:1135–40. 10.1086/502680 16575731

[25] Church D Cerutti L Gürtler A Griener T Zelazny A Emler S. Performance and application of 16s RRNA gene cycle sequencing for routine identification of bacteria in the clinical microbiology laboratory. *Clin Microbiol Rev.* (2020) 33:e53–19. 10.1128/cmr.00053-19 32907806 PMC7484979

[26] Fernández Vecilla D Roche Matheus M Calvo Muro F Iglesias Hidalgo G Díaz de Tuesta Del Arco JL. Identification of curved gram-negative rods by maldi-tof mass spectrometer in a patient with fournier’S gangrene. A bacteremia caused by desulfovibrio desulfuricans and *Escherichia Coli*. *Rev Esp Quimioter.* (2023) 36:629–31. 10.37201/req/026.2023 37767548 PMC10710683

[27] Duan H Li X Mei A Li P Liu Y Li X The diagnostic value of metagenomic next-generation sequencing in infectious diseases. *BMC Infect Dis.* (2021) 21:62. 10.1186/s12879-020-05746-5 33435894 PMC7805029

[28] Han D Li Z Li R Tan P Zhang R Li J. Mngs in clinical microbiology laboratories: On the road to maturity. *Crit Rev Microbiol.* (2019) 45:668–85. 10.1080/1040841x.2019.1681933 31691607

