# Supplementary material for: Severe pneumonia with empyema due to multiple anaerobic infections: case report and literature review
Source: Front Med (Lausanne). 2024 Aug 14;11:1435823. doi: 10.3389/fmed.2024.1435823 (PMC11349742; doi:10.3389/fmed.2024.1435823)
Supplement: Supplementary file 1 [file Data_Sheet_1.docx]

Supplementary Material

**Supplementary Table 1.** SOFA scores of the patiens throughout the clinical course.

| **Length of hospital stay** | **SOFA Score** |
| --- | --- |
| D1 (Feb-26) | 17 |
| D3 | 18 |
| D5 | 15 |
| D7 | 10 |
| D9 | 13 |
| D11 | 12 |
| D13 | 11 |
| D15 | 12 |
| D17 | 13 |
| D19 | 11 |
| D21 | 11 |
| D23 | 12 |
| D25 | 10 |

**Supplementary Table 2.** The relative abundance of the pathogens identified by BALF, Pleural effusion and blood mNGS.

| **Sample** | **Pathogens** | **Relative abundance at species level** |
| --- | --- | --- |
| BALF | *Parvimonas micra* | 18.20% |
|  | *Peptostreptococcus stomatis* | 18.10% |
|  | *Olsenella uli* | 13.90% |
|  | *Slackia exigua* | 8.30% |
|  | *Bacteroides heparinolyticus* | 6.00% |
|  | *Streptococcus anginosus* | 5.60% |
|  | *Gemella morbillorum* | 5.60% |
|  | *Porphyromonas endodontalis* | 4.00% |
|  | *Streptococcus pneumoniae* | 0.90% |
|  | *Staphylococcus aureus* | 0.01% |
| Pleural effusion | *Peptostreptococcus stomatis* | 29.30% |
|  | *Slackia exigua* | 23.00% |
|  | *Olsenella uli* | 22.90% |
|  | *Filifactor alocis* | 7.70% |
|  | *Porphyromonas endodontalis* | 6.00% |
|  | *Bacteroides heparinolyticus* | 3.00% |
|  | *Dialister pneumosintes* | 2.50% |
|  | *Prevotella baroniae* | 1.50% |
|  | *Alloprevotella tannerae* | 0.45% |
|  | *Fusobacterium nucleatum* | 0.09% |
|  | *Campylobacter rectus* | 0.09% |
| Blood | *Bacteroides heparinolyticus* | 2.40% |
|  | *Alloprevotella tannerae* | 1.80% |
|  | *Porphyromonas endodontalis* | 1.80% |
|  | *Streptococcus pneumoniae* | 0.80% |

Note: Relative abundance refers to the proportion of distribution of a microbial species sequence in the corresponding species (usually divided into bacteria, fungi, viruses, and parasitic insects) after the removal of the host sequence.

**Supplementary Table 3.** Clinical data of patients diagnosed with anaerobic pleural empyema.

| **ID** | **Year** | **Sex** | **Age** | **Underlying diseases** | **Initial symptoms** | **radiographic appearance** | **Sample** | **Confirmed method** | **Species** | **surgery** | **Mechanical ventilation** | **Therapy** | **Outcome** |
| --- | --- | --- | --- | --- | --- | --- | --- | --- | --- | --- | --- | --- | --- |
| 1 | 2024 (1) | male | 50 | poly-substance use disorder, hyperlipidemia, smoking history | chest pain, cough | hazy opacification of the right lower lung field; areas of lucency in the medial margin of the consolidation may correspond to the aerated lung; necrosis has a similar appearance | body fluid, pleural fluid, surgical swab | culture | Parvimonas micra, COVID-19 | right postero-lateral open thoracotomy, total lung decortication, wedge resection of the right lower and middle lobe, pneumonolysis, and mechanical pleurodesis | NIPPV | levofloxacin, vancomycin, and Zosyn | recovery |
| 2 | 2024 (2) | male | 39 | bronchial asthma and a mild form of periodontal disease, smoking and drinking history | back pain | a mass with pleural thickening and an internal low-attenuation area at the site of the previous empyema cavity | pleural fluid | culture | Actinomyces naeslundii | thoracoscopic surgery | no | Amoxicillin/Clavulanate, Ampicillin/Sulbactam, Amoxicillin/Clavulanate | recovery |
| 3 | 2024 (3) | male | 77 | chronic obstructive pulmonary disease, chronic kidney disease, and anemia, smoking history | dyspnea, cough, weight loss, decreased appetite, and night sweats | nodular opacities with bilateral pleural effusions | empyema | mNGS | Pseudopropionibacterium propionicum | no | no | ceftriaxone and minocycline, minocycline | died |
| 4 | 2023 (4) | male | 28 | pulmonary tuberculosis | fever, weight loss, dyspnea | an extensive empyema in the left hemithorax | empyema | MALDI-TOF MS | Parvimonas micra | no | no | piperacillin/tazobactam | recovery |
| 5 | 2023 (5) | female | 51 | smoking history | chest pain | a large right-sided fluid collection | Tissue | culture | Peptostreptococcus, Veillonella | VATS | no | intravenous ampicillin-sulbactam and ertapenem | recovery |
| 6 | 2023 (6) | male | 60 | hypertension, a mood disorder, dental surgery, smoking and drinking history | cough, dyspnea, appetite | a massive right-sided pleural effusion, with gas components suggesting a possible empyema | pleural fluid | cultures, fluid analysis, and cytology | Tannerella forsythia, Fusobacterium nucleatum， Actinomyces | thoracostomy, VATS | no | ampicillin-sulbactam, amoxicillin-clavulanic acid | recovery |
| 7 | 2023 (7) | male | 37 | bronchial asthma | right-sided dull aching pain in the subcoastal region, high-grade fever with chills and shortness of breath | moderate effusion accompanied by underlying lung collapse, multiple enlarged mediastinal lymph nodes were noted in pre-vascular, sub-carinal, sub-aortic, paratracheal, paraaortic, and para-esophageal stations | blood, pleural fluid | culture | Prevotella buccae | no | no | intercostal drain | recovery |
| 8 | 2023 (8) | male | 49 | tobacco and alcohol consumption, periodontitis | cough, sputum, dyspnea | infectious lesions in both lungs, the giant cavity of the left lung with gas and fluid levels | pus, peripheral blood | mNGS, culture | Porphyromonas gingivalis | no | MV with endotracheal intubation | imipenem | recovery |
| 9 | 2023 (9) | male | 8 | Down's syndrome | fever and cough | pleural effusion and lung abscess | sputum, Pleural effussion | mNGS | Parvimonas micra, Porphyromonas gingivalis | one of the children underwent lung necrosis tissue resection | one required MV, three with low-flow oxygen support | metronidazole and piperacillin tazobactam | recovery |
| 10 |  | male | 13 | Cerebral palsy |  |  |  |  |  |  |  |  |  |
| 11 |  | male | 11 | Cerebral palsy |  |  |  |  |  |  |  |  |  |
| 12 |  | female | 9 | Hypophrenia |  |  |  |  |  |  |  |  |  |
| 13 | 2023 (10) | unknown | 4 | sickle cell anemia | Fever, cough, and purulent expectoration | condensation in the right upper lobe | broncho-aspirate, bronchial brushing and BALF | Culture, 16S rRNA | Lautropia mirabillis, Prevotella oris | no | no | Cefotaxime and vancomycin | recovery |
| 14 |  | unknown | 13 | Glanzmann’s thrombasthenia and allergic asthma | left flank pain radiating to the shoulder and respiratory distress | condensation in the left lung base | pleural effusion | 16S rRNA | Prevotella oris | no | no | Cefotaxime and vancomycin and clindamycin | recovery |
| 15 | 2022 (11) | Male | 51 | Smocking and drinking history | right-sided chest pain | mild haziness in the right lung, right sided pleural effusion | pleural pus | Culture, MALDI TOF MS | Prevotella oris | no | no | metronidazole | recovery |
| 16 | 2022 (12) | male | 48 | drinking history | erythema and painful lesions on the right side of the chest, dyspnea, weakness, and weight loss | multiple voids, wall thickening and pleural effusion, right chest abscess | pleural fluid | culture, MALDI-TOF MS | Campylobacter rectus | no | no | imipenem, amoxicillin clavulanic acid and metronidazole | recovery |
| 17 | 2022 (13) | female | 74 | gastric cancer, periodontitis | regrowth of the chest wall mass with tenderness | a subcutaneous abscess with a diameter of 9 cm extending from a right lower subpleural lung abscess with a diameter of 8 cm accompanied by empyema | pus | culture, 16 S rRNA PCR assay | Porphyromonas gingivalis | the subcutaneous abscess with debridement | no | ampicillin/sulbactam, amoxicillin/clavulanate | recovery |
| 18 | 2021 (14) | male | 52 | smoking history, drinking history | shortness of breath, cough | a massive pleural effusion on the left with mediastinal lymphadenopathy and shift to the right side | pleural effusion fluid, septae biopsies | MALDI-TOF MS | Actinomyces meyeri | minithoracotomy with empyema evacuation, complete parietal pleurectomy, decortications of the left upper and lower lobes | NIV | amoxicillin/clavulanic acid, amoxicillin | recovery |
| 19 | 2021 (15) | male | 33 | obesity, T2DM, obstructive sleep apnea | chest pain, inspiration, cough | a large, loculated pleural effusion with partial collapse of the right upper and lower lobes | pleural fluid | gram stain | Fusobacterium nucleatum, Actinomyces turicensis | no | no | ampicillin/sulbactam, amoxicillin/clavulanic acid | recovery |
| 20 | 2021 (16) | male | 78 | none | dyspnea and chills | a large and multiloculated pleural effusion with an air bubble on the right side | pleural effusion | culture and 16S rRNA gene | Streptococcus constellatus | no | no | ceftriaxone and clindamycin, cefpodoxime and clindamycin | recovery |
| 21 | 2021 (17) | Female | 65 | diabetes mellitus | fatigue, appetite loss, cough, and dyspnea | extensive pleural effusion with slight bubbles and air in the left pleural cavity, which caused the collapse of the left lung and mediastinal shift to the right | pus | smear microscopy | anaerobic bacteria | no | no | sulbactam, ampicillin | recovery |
| 22 | 2020 (18) | female | 66 | grade 2 bronchogenic adenocarcinoma | cough, shortness of breath and dyspnea | left upper lung lobe mass about 50×52×60 mm impressive of bronchogenic carcinoma, with mild pleural effusion on the left side | pus | culture | Salmonella | No | No | levofloxacin, ceftazidime, clindamycin | recovery |
| 23 | 2019 (19) | male | 75 | tobacco abuse and depression | fatigue, coughing up blood-tinged sputum | a large, lower, right-sided loculated effusion | blood, pleural fluid | culture | Streptococcus gordonii | VATS | no | ampicillin/sulbactam | / |
| 24 | 2018 (20) | female | 40 | hypertension, asthma, smoking history, drinking history | cough, sputum, fever, chills, watery diarrhea, and vomiting | left pulmonary abscess with pleural effusion | blood, pleural fluid | culture | Eggerthia catenaformis, Fusobacterium nucleatum, Parvimonas micra | no | MV | amoxicillin-clavulanate for 6 weeks | recovery |
| 25 | 2018 (21) | male | 69 | chronic renal failure, atherosclerosis and chronic obstructive pulmonary disease, drinking history | back pain, shortness of breath | an empyema and a large broncho-pleural fistula complicating a necrotic pneumonia in the inferior lobe of the right lung | pleural liquid | culture and MALDI-TOF MS | Campylobacter rectus | no | no | / | died |
| 26 | 2017 (22) | male | 75 | smoking history, rheumatoid arthritis, T2DM, thoracoscopy and talc pleurodesis | breathlessness, malaise, cough, fevers | a right‐sided hydropneumothorax and a stable left‐sided effusion | pleural fluid | culture, 16S rRNA PCR testing | Propionibacterium acnes | surgical decortication | no | piperacillin/tazobactam | recovery |
| 27 | 2017 (23) | female | 65 | bronchiectasis | cough, phlegm | a cavitary lesion in the left upper lobe superior lingular segment | blood, pleural effusion | 16 S rRNA PCR | Campylobacter curvus | no | no | ampicillin/sulbactam and clindamycin | recovery |
| 28 | 2016 (24) | male | 24 | smoking history, have a wisdom tooth extracted | fever, dyspnea, severe fatigue | right pleural effusion, no embolism or pulmonary abscess | blood | culture | Fusobacterium necrophorum | no | no | Amoxicillin/clavulanic acid, and metronidazole | recovery |
| 29 | 2014 (25) | male | 76 | hypertension | fever and chest pain | left encapsulated pleural effusion, pleural thickening and adhesion | pleural pus，sputum, blood, bronchoalveolar lavage fluid and pleural fluid | 16S rDNA sequence analysis | Prevotella spp | no | no | Imipenem, metronidazole | recovery |
| 30 | 2012 (26) | male | 68 | adenocarcinoma of the sigmoid colon, recurrent urinary tract infections, nephrolithiasis, and hydronephrosis | breathlessness, cough, left-sided loin pains | a left-sided perinephric collection and a large pleural effusion that had gas-containing areas consistent with an empyema | pleural fluid, urine | culture | Escherichia coli, Enterococcus faecalis | no | endotracheal intubation and MV | piperacillin/tazobactam, metronidazole, amoxicillin, ciprofloxacin. | recovery |
| 31 | 2011 (27) | male | 22 | no | Sore throat, fever, difficulty swallowing | Lung consolidation, lung infiltration, pleural effusion, Gas-liquid plane on the right | pleural fluid | culture | Fusobacterium necrophorum | endoscopic treatment | no | meropenem, clarithromycin, linezolid | recovery |
| 32 | 2008 (28) | male | 38 | / | sore throat, shortness of breath, and high fever | left peritonsillar abscess formation, occlusion of the left internal jugular vein with inflammatory wall thickening and perijugular soft tissue infiltration, pulmonary abscesses, and bilateral pleural effusions | blood | culture | Fusobacterium necrophorum | staged decortication of bilateral empyema | no | clindamycin | recovery |
| 33 | 2006 (29) | Female | 19 | anorexia nervosa | cough, fever, malaise, left chest pain | massive pleural effusion and gas in the left pleural cavity, causing ashift of the mediastinum to the right side | pleural fluids | culture | Peptostreptococcus | no | no | clindamycin, meropenem | recovery |
| 34 | 2006 (30) | male | 59 | cerebrovascular accident | fever | right submassive pleural effusion | pleural fluid | culture | Streptococcus constellatusas, Prevotella intermedia, Fusobacterium urealyticus | no | no | ceftriaxone, clindamycin | recovery |
| 35 | 2001 (31) | male | 54 | alcoholic cirrhosis | fever and shortness of breath | right empyema and collapse-consolidation of the lower lobe of the right lung | blood, pleural fluid | culture,16S rRNA gene sequence | Laribacter hongkongensis gen. nov., sp. nov. | no | no | cefuroxime, netilmicin | recovery |
| 36 | 2000 (32) | Male | 62 | chronic sinusitis, smoking and drinking history | chronic sputum, dyspnea, and weakness | severe mediastinal shift and left lung collapse due to the accumulation of fluid and gas in the left pleural space | pleural effusion | culture | Bacteroides spp. | no | no | imipenem/cilastatin sodium, clindamycin | recovery |
| 37 | 2000 (33) | male | 50 | spontaneous pneumothorax, drinking and smoking history | disturbance of consciousness, chest pain | massive pleural effusion with air fluid level and deviation of mediastinum to the left | pleural fluid | culture | Bacteroides fragilis, Citrobacter koseri, Enterococcus species | no | no | imipenem/cilastatin, amikacin | recovery |
| 38 | 1998 (34) | male | 68 | malaria, rheumatic fever, emphysema, myocardial infarcts angina, drinking and smoking history | rigors, right hypochondrial pain and ankle swelling | signs of a right pleural effusion | aspirate, empyema | cultures | Clostridium tetani, Fusobacterium mortiferum | thoracotomy and drainage of the effusion | no | penicillin | died |
| 39 | 1996 (35) | Male | 68 | anorectic | weakness, malaise, weight loss | a large mass along the left side of the heart which appeared to extend into the left lung with consilidation and bilateral pleural effusions | abscess | culture | Peptostreptococcus anaerobius | pulmonary artery catheter insertion and thoracotomy with chest tube drainage | / | / | / |
| 40 | 1989 (36) | male | 41 | chronic alcohol abuser | clouding of consciousness | right-side pneumonia | fluid, blood | culture | Gardnerella vaginalis, Streptococcus milleri, Bacteroides oralis, Peptiostreptococcussp., Peptococcus sp., Veillonella sp., Haemophilus parainjfluenzae,Streptococcus milleri, Neisseria sicca. ect. | no | no | ceftazidime, penicillin, minocycline, metronidazole, clindamycin, ampicillin | died |

|  |
| --- |

NIV: non-invasive ventilation; NIPPV: noninvasive positive pressure **ventilation**; MV: Mechanical ventilation; VATS: video-assisted thoracoscopic surgery.

**Reference**

1. Gumbs S, Kwentoh I, Atiku E, Gikunda W, Safavi A. Parvimonas Micra: A Rare Cause of Pleural Empyema with Covid-19 Co-Infection. *Cureus* (2024) 16(1):e51998. doi: 10.7759/cureus.51998.

2. Matsubayashi Y, Mizuno K, Yamashita T, Asai K. A Case of Thoracic Empyema Caused by Actinomyces Naeslundii. *American Journal of Case Reports* (2024) 25:e943030. doi: 10.12659/ajcr.943030.

3. Babar S, Liu E, Kaur S, Hussain J, Danaher PJ, Anstead GM. Pseudopropionibacterium Propionicum as a Cause of Empyema; a Diagnosis with Next-Generation Sequencing. *Pathogens* (2024) 13(2):165. doi: 10.3390/pathogens13020165.

4. Vilcarromero S, Small M, Lizarzaburu A, Rivadeneyra-Rodriguez A. Pleural Empyema by Parvimonas Micra in an Immunocompetent Patient: A Case Report. *Rev Peru Med Exp Salud Publica* (2023) 40(1):99-104. doi: 10.17843/rpmesp.2023.401.11956.

5. Martinez K, Mangat GK, Sherwani N, Glover DM, Silver Md M. Veillonella Intrapulmonary Abscess with Empyema. *Cureus* (2023) 15(9):e45210. doi: 10.7759/cureus.45210.

6. Stewart CE, McCafferty A, Sherertz R. Tannerella Forsythia, Fusobacterium Nucleatum, and Suspected Actinomyces Causing Massive Empyema: A Case Report. *Cureus* (2023) 15(11):e48117. doi: 10.7759/cureus.48117.

7. Patel S, Hanfe H, Khurana AK. Unilateral Complicated Pleural Empyema in a Patient with Bronchial Asthma Due to Clindamycin-Resistant Prevotella Buccae. *Archive of Clinical Cases* (2023) 10(4):150-2. doi: 10.22551/2023.41.1004.10263.

8. Sha J, Shao J, Lu S, Yao W, Deng Y, Chen J, et al. Pyopneumothorax with Bronchopleural Fistula Due to Pulmonary Infection Caused by Porphyromonas Gingivalis in a Patient with Periodontitis. *The Clinical Respiratory Journal* (2023) 17(9):962-5. doi: 10.1111/crj.13684.

9. Zhijun L, Wenhai Y, Peibin Z, Qingming L. Pediatric Pulmonary Infection Caused by Oral Obligate Anaerobes: Case Series. *Frontiers in Pediatrics* (2023) 11:1226706. doi: 10.3389/fped.2023.1226706.

10. Fernández Vecilla D, Roche Matheus MP, Iglesias Hidalgo G, Ugalde Zárraga E, Unzaga Barañano MJ, Díaz de Tuesta del Arco JL. Two Cases of Prevotella Oris Causing Serious Pleuropulmonary Infections. *Revista Española de Quimioterapia* (2023) 36(4):439-41. doi: 10.37201/req/001.2023.

11. Viswanath LS, Gunalan A, Jamir I, S B, S A, K A, et al. Prevotella Oris: A Lesser Known Etiological Agent of Pleural Effusion. *Anaerobe* (2022) 78:102644. doi: 10.1016/j.anaerobe.2022.102644.

12. Barberis C, Florencia Veiga M, Tolosa D, Vay C, Schuarzberg P. Empyema Necessitatis Caused by Campylobacterrectus. Rapid Identification by Maldi-Tof Ms. *Revista Argentina de Microbiología* (2022) 54(4):305-8. doi: 10.1016/j.ram.2022.03.001.

13. Tanaka A, Kogami M, Nagatomo Y, Takeda Y, Kanzawa H, Kawaguchi Y, et al. Subcutaneous Abscess Due to Empyema Necessitans Caused by Porphyromonas Gingivalis in a Patient with Periodontitis. *IDCases* (2022) 27. doi: 10.1016/j.idcr.2022.e01458.

14. Hoheisel A, Herrmann MJ, Kassi E, Hojski A, Tamm M, Jahn K. Actinomyces Meyeri Pleural Empyema: A Case Report. *IDCases* (2021) 26:e01278. doi: 10.1016/j.idcr.2021.e01278.

15. Johnson SW, Billatos E. Polymicrobial Empyema; a Novel Case of Actinomyces Turicensis. *Respiratory Medicine Case Reports* (2021) 32:101365. doi: 10.1016/j.rmcr.2021.101365.

16. Lee YJ, Lee J, Kwon BS, Kim Y. An Empyema Caused by Streptococcus Constellatus in an Older Immunocompetent Patient. *Medicine* (2021) 100(45):e27893. doi: 10.1097/md.0000000000027893.

17. Kanai O, Fujita K, Okamura M, Nakatani K, Mio T. Afebrile Tension Pyopneumothorax Due to Anaerobic Bacteria: Fistula or Gas Production? *Respiratory Medicine Case Reports* (2021) 32:101372. doi: 10.1016/j.rmcr.2021.101372.

18. Samir Abdelhafiz A, Wassef M, Alorabi M. Pleural Empyema Due to Salmonella in a Patient with Bronchogenic Carcinoma: The First Case Report from a Cancer Hospital in Egypt. *Access microbiology* (2020) 2(9):acmi000151. doi: 10.1099/acmi.0.000151.

19. Farooq H, Mohammad T, Farooq A, Mohammad Q. Streptococcus Gordonii Empyema: A Rare Presentation of Streptococcus Gordonii Infection. *Cureus* (2019) 11(5):e4611. doi: 10.7759/cureus.4611.

20. Duport P, Miltgen G, Kebbabi C, Belmonte O, Coolen-Allou N, Allyn J, et al. First Case of Pleural Empyema and Pulmonary Abscess Caused by Eggerthia Catenaformis. *Anaerobe* (2018) 50:9-11. doi: 10.1016/j.anaerobe.2018.01.006.

21. Noël A, Verroken A, Belkhir L, Rodriguez-Villalobos H. Fatal Thoracic Empyema Involving Campylobacter Rectus : A Case Report. *Anaerobe* (2018) 49:95-8. doi: 10.1016/j.anaerobe.2017.12.014.

22. Lawrence H, Moore T, Webb K, Lim WS. Propionibacterium Acnes Pleural Empyema Following Medical Thoracoscopy. *Respirology Case Reports* (2017) 5(5):e00249. doi: 10.1002/rcr2.249.

23. Horio Y, Shiraishi Y, Watanabe N, Inoue S, Imanishi T, Asano K. Empyema Associated with Campylobacter Curvus Infection. *Respirology Case Reports* (2017) 5(4):e00234. doi: 10.1002/rcr2.234.

24. Habert P, Tazi-Mezalek R, Guinde J, Martinez S, Laroumagne S, Astoul P, et al. Pleuro-Pneumonia Revealing Septic Thrombophlebitis of the Jugular Vein: Think About the Lemierre's Syndrome. *Revue des Maladies Respiratoires* (2016) 33(1):72-7. doi: 10.1016/j.rmr.2015.05.006.

25. Zhou H, Shen Y, Shen Q, Zhou J. Thoracic Empyema Caused by Prevotella Spp. Diagnosed Using 16s Rdna Sequence Analysis. *The Clinical Respiratory Journal* (2014) 9(1):121-4. doi: 10.1111/crj.12106.

26. Jones GH, Kalaher HR, Misra N, Curtis J, Parker RJ. Empyema and Respiratory Failure Secondary to Nephropleural Fistula Caused by Chronic Urinary Tract Infection: A Case Report. *Case Reports in Pulmonology* (2012) 2012:1-5. doi: 10.1155/2012/595402.

27. Gülmez D, Alp S, Topeli İskit A, Akova M, Hasçelik G. [Pneumonia Caused by Fusobacterium Necrophorum: Is Lemierre Syndrome Still Current?]. *Mikrobiyol Bul* (2011) 45(4):729-34.

28. Escher R, Haltmeier S, von Steiger N, Dutly AE, Arnold A, Kickuth R, et al. Advanced Lemierre Syndrome Requiring Surgery. *Infection* (2008) 36(5):495-6. doi: 10.1007/s15010-008-8216-7.

29. Kikuchi N, Nomura A, Endo T, Sekizawa K. Anaerobic Bacterial Empyema Accompanying Intrathoracic Gas Formation in Anorexia Nervosa. *Int J Eat Disord* (2006) 39(7):621-3. doi: 10.1002/eat.20275.

30. Díaz Peromingo JA, Sánchez Leira J, García Suárez F, Padín Paz E, Saborido Froján J. Streptococcus Constellatusas a Causative Agent of Empyema. Report of One Case. *Revista medica de Chile* (2006) 134(8):1030-2. doi: 10.4067/s0034-98872006000800013.

31. Yuen K-Y, Woo PCY, Teng JLL, Leung K-W, Wong MKM, Lau SKP. Laribacter Hongkongensis Gen. Nov., Sp. Nov., a Novel Gram-Negative Bacterium Isolated from a Cirrhotic Patient with Bacteremia and Empyema. *Journal of Clinical Microbiology* (2001) 39(12):4227-32. doi: 10.1128/jcm.39.12.4227-4232.2001.

32. Endo T, Saito T, Ohse H, Nakayama M, Watanabe S, Sekizawa K, et al. Anaerobic Bacillus Pyothorax with the Production of Gas and Severe Mediastinal Shift. *Nihon Kokyuki Gakkai zasshi* (2000) 38(1):45-9.

33. Kuze N, Nishizaka Y, Okamoto K, Wakayama T, Imanaka M, Kubo Y, et al. Empyema Thoracis Accompanied by Hyperammonemic Encephalopathy. *Nihon Kokyuki Gakkai zasshi* (2000) 38(2):117-21.

34. Mayall BC, Snashall EA, Peel MM. Isolation of Clostridium Tetani from Anaerobic Empyema. *Pathology* (1998) 30(4):402-4. doi: 10.1080/00313029800169716.

35. Colleran JA, Ballo MS, Papademetriou V, Lu D, Fletcher RD. Regional Cardiac Compression Due to a Large Anaerobic Bacterial Empyema. *Clinical cardiology* (1996) 19(4):332-4. doi: 10.1002/clc.4960190410.

36. Legrand JC, Alewaeters A, Leenaerts L, Gilbert P, Labbe M, Glupczynski Y. Gardnerella Vaginalis Bacteremia from Pulmonary Abscess in a Male Alcohol Abuser. *Journal of clinical microbiology* (1989) 27(5):1132-4. doi: 10.1128/jcm.27.5.1132-1134.1989.
